# Supplementary material for: Evaluating Traditional, Deep Learning and Subfield Methods for Automatically Segmenting the Hippocampus From MRI
Source: Hum Brain Mapp. 2025 Mar 27;46(5):e70200. doi: 10.1002/hbm.70200 (PMC11947432; doi:10.1002/hbm.70200)
Supplement: Supplementary file 1 — Data S1. [file HBM-46-e70200-s001.docx]

**Evaluating Traditional, Deep Learning, and Subfield Methods for Automatically Segmenting the Hippocampus from MRI

Supplementary Material**

Table S1: Mean volume, mean volume similarity, mean Dice coefficient and mean 95% Hausdorff distance for AD subjects from the ADNI HarP dataset, split across field strength and scanner manufacturer

| Group: AD | **FreeSurfer** | **SynthSeg** | **FastSurfer** | **FIRST** | **Hippodeep** | | | **FreeSurfer-Subfields** | **HippUnfold** | **HSF** |
| --- | --- | --- | --- | --- | --- | --- | --- | --- | --- | --- |
| **Volume (mm^3^)** | | | | | | |  |  |  |  |
| *Field Strength* |  |  |  |  |  | | |  |  |  |
| 1.5T | 3,091 (708) | 3,217 (596) | 3,090 (644) | 3,115 (585) | 2,242 (655) | | | 2,650 (519) | 2,088 (447) | 4,130 (542) |
| 3T | 3,011 (504) | 3,034 (567) | 2,975 (536) | 2,805 (579) | 2,094 (496) | | | 2,537 (456) | 1,997 (433) | 4,127 (751) |
| *Manufacturer* |  |  |  |  |  | | |  |  |  |
| GE | 2,954 (467) | 3,160 (426) | 3,046 (432) | 3,058 (443) | 2,172 (415) | | | 2,534 (348) | 2,046 (354) | 4,056 (540) |
| Philips | 3,249 (685) | 3,197 (688) | 3,201 (731) | 2,966 (787) | 2,270 (738) | | | 2,749 (555) | 2,107 (508) | 4,294 (721) |
| Siemens | 2,968 (625) | 3,043 (620) | 2,892 (568) | 2,875 (550) | 2,088 (571) | | | 2,523 (520) | 1,989 (452) | 4,062 (675) |
| **Volume Similarity** | | | | | |  |  |  |  |  |
| *Field Strength* |  |  |  |  |  | | |  |  |  |
| 1.5T | 0.21 (0.10) | 0.26 (0.09) | 0.22 (0.08) | 0.23 (0.12) | -0.12 (0.15) | | | 0.07 (0.09) | -0.17 (0.10) | 0.51 (0.17) |
| 3T | 0.25 (0.12) | 0.26 (0.12) | 0.24 (0.09) | 0.18 (0.14) | -0.11 (0.11) | | | 0.09 (0.10) | -0.16 (0.14) | 0.55 (0.18) |
| *Manufacturer* |  |  |  |  |  | | |  |  |  |
| GE | 0.20 (0.15) | 0.27 (0.10) | 0.23 (0.09) | 0.24 (0.14) | -0.11 (0.12) | | | 0.05 (0.10) | -0.16 (0.12) | 0.51 (0.16) |
| Philips | 0.24 (0.08) | 0.22 (0.10) | 0.22 (0.06) | 0.13 (0.11) | -0.15 (0.13) | | | 0.07 (0.08) | -0.20 (0.14) | 0.51 (0.19) |
| Siemens | 0.26 (0.10) | 0.29 (0.10) | 0.24 (0.10) | 0.23 (0.13) | -0.11 (0.15) | | | 0.10 (0.10) | -0.14 (0.10) | 0.56 (0.19) |
| **Dice Coefficient** | | | | | | |  |  |  |  |
| *Field Strength* |  |  |  |  |  | | |  |  |  |
| 1.5T | 0.67 (0.05) | 0.77 (0.04) | 0.69 (0.04) | 0.78 (0.04) | 0.79 (0.04) | | | 0.69 (0.05) | 0.70 (0.11) | 0.71 (0.09) |
| 3T | 0.67 (0.04) | 0.78 (0.05) | 0.68 (0.04) | 0.79 (0.03) | 0.81 (0.02) | | | 0.69 (0.03) | 0.73 (0.07) | 0.69 (0.09) |
| *Manufacturer* |  |  |  |  |  | | |  |  |  |
| GE | 0.66 (0.06) | 0.77 (0.04) | 0.69 (0.04) | 0.78 (0.03) | 0.79 (0.03) | | | 0.68 (0.05) | 0.73 (0.04) | 0.71 (0.07) |
| Philips | 0.68 (0.04) | 0.78 (0.05) | 0.68 (0.04) | 0.80 (0.03) | 0.81 (0.03) | | | 0.70 (0.03) | 0.70 (0.11) | 0.71 (0.09) |
| Siemens | 0.67 (0.04) | 0.77 (0.04) | 0.68 (0.04) | 0.78 (0.04) | 0.80 (0.04) | | | 0.69 (0.04) | 0.70 (0.10) | 0.68 (0.10) |
| **95% Hausdorff Distance (mm)** | | | | | | |  |  |  |  |
| *Field Strength* |  |  |  |  |  | | |  |  |  |
| 1.5T | 2.59 (0.79) | 1.75 (0.30) | 2.20 (0.27) | 1.72 (0.33) | 1.52 (0.32) | | | 2.00 (0.49) | 2.64 (1.52) | 2.64 (1.27) |
| 3T | 2.64 (0.52) | 1.68 (0.54) | 2.24 (0.26) | 1.59 (0.28) | 1.35 (0.21) | | | 1.74 (0.29) | 2.28 (1.09) | 2.71 (1.26) |
| *Manufacturer* |  |  |  |  |  | | |  |  |  |
| GE | 2.61 (0.67) | 1.71 (0.29) | 2.19 (0.18) | 1.73 (0.32) | 1.46 (0.24) | | | 2.00 (0.57) | 2.21 (0.60) | 2.52 (0.76) |
| Philips | 2.56 (0.75) | 1.66 (0.56) | 2.23 (0.31) | 1.52 (0.29) | 1.37 (0.18) | | | 1.86 (0.37) | 2.49 (1.48) | 2.55 (1.42) |
| Siemens | 2.65 (0.61) | 1.77 (0.44) | 2.24 (0.29) | 1.69 (0.30) | 1.46 (0.36) | | | 1.78 (0.27) | 2.63 (1.59) | 2.90 (1.44) |

Table S2: Mean volume, mean volume similarity, mean Dice coefficient and mean 95% Hausdorff distance for MCI subjects from the ADNI HarP dataset, split across field strength and scanner manufacturer

| Group: MCI | **FreeSurfer** | **SynthSeg** | **FastSurfer** | **FIRST** | | **Hippodeep** | **FreeSurfer-Subfields** | **HippUnfold** | **HSF** |
| --- | --- | --- | --- | --- | --- | --- | --- | --- | --- |
| **Volume (mm^3^)** | | | | |  |  |  |  |  |
| *Field Strength* |  |  |  |  | |  |  |  |  |
| 1.5T | 3,313 (549) | 3,450 (563) | 3,333 (570) | 3,281 (562) | | 2,511 (523) | 2,787 (508) | 2,229 (409) | 4,080 (470) |
| 3T | 3,461 (486) | 3,439 (468) | 3,443 (427) | 3,164 (429) | | 2,490 (401) | 3,009 (392) | 2,289 (362) | 4,170 (638) |
| *Manufacturer* |  |  |  |  | |  |  |  |  |
| GE | 3,343 (453) | 3,504 (462) | 3,370 (462) | 3,326 (485) | | 2,544 (421) | 2,843 (475) | 2,307 (394) | 4,079 (493) |
| Philips | 3,411 (678) | 3,398 (645) | 3,383 (636) | 3,196 (572) | | 2,483 (578) | 2,893 (577) | 2,254 (475) | 4,249 (587) |
| Siemens | 3,381 (397) | 3,432 (433) | 3,402 (418) | 3,157 (451) | | 2,477 (397) | 2,931 (354) | 2,211 (277) | 4,038 (571) |
| **Volume Similarity** | | | | |  |  |  |  |  |
| *Field Strength* |  |  |  |  | |  |  |  |  |
| 1.5T | 0.20 (0.07) | 0.25 (0.06) | 0.22 (0.06) | 0.20 (0.08) | | -0.07 (0.08) | 0.05 (0.07) | -0.18 (0.06) | 0.42 (0.11) |
| 3T | 0.25 (0.08) | 0.26 (0.07) | 0.26 (0.08) | 0.17 (0.10) | | -0.07 (0.08) | 0.10 (0.08) | -0.16 (0.08) | 0.44 (0.10) |
| *Manufacturer* |  |  |  |  | |  |  |  |  |
| GE | 0.20 (0.07) | 0.26 (0.07) | 0.23 (0.07) | 0.21 (0.09) | | -0.06 (0.08) | 0.05 (0.07) | -0.15 (0.06) | 0.41 (0.10) |
| Philips | 0.23 (0.09) | 0.22 (0.07) | 0.22 (0.08) | 0.17 (0.11) | | -0.10 (0.09) | 0.06 (0.10) | -0.19 (0.09) | 0.45 (0.13) |
| Siemens | 0.25 (0.06) | 0.27 (0.05) | 0.26 (0.05) | 0.19 (0.07) | | -0.06 (0.06) | 0.11 (0.06) | -0.16 (0.05) | 0.43 (0.10) |
| **Dice Coefficient** | | | | |  |  |  |  |  |
| *Field Strength* |  |  |  |  | |  |  |  |  |
| 1.5T | 0.69 (0.04) | 0.79 (0.03) | 0.70 (0.05) | 0.80 (0.03) | | 0.82 (0.03) | 0.71 (0.04) | 0.75 (0.05) | 0.75 (0.06) |
| 3T | 0.69 (0.03) | 0.80 (0.03) | 0.70 (0.04) | 0.81 (0.03) | | 0.82 (0.03) | 0.71 (0.03) | 0.77 (0.05) | 0.75 (0.05) |
| *Manufacturer* |  |  |  |  | |  |  |  |  |
| GE | 0.70 (0.03) | 0.79 (0.03) | 0.70 (0.04) | 0.80 (0.03) | | 0.82 (0.02) | 0.71 (0.03) | 0.77 (0.03) | 0.76 (0.04) |
| Philips | 0.70 (0.04) | 0.80 (0.03) | 0.71 (0.05) | 0.80 (0.03) | | 0.82 (0.04) | 0.71 (0.04) | 0.74 (0.07) | 0.74 (0.07) |
| Siemens | 0.68 (0.04) | 0.79 (0.03) | 0.69 (0.04) | 0.80 (0.03) | | 0.82 (0.02) | 0.70 (0.03) | 0.77 (0.03) | 0.75 (0.05) |
| **95% Hausdorff Distance (mm)** | | | | |  |  |  |  |  |
| *Field Strength* |  |  |  |  | |  |  |  |  |
| 1.5T | 2.40 (0.47) | 1.76 (0.28) | 2.21 (0.26) | 1.72 (0.28) | | 1.41 (0.21) | 1.86 (0.32) | 2.06 (0.93) | 2.10 (0.58) |
| 3T | 2.49 (0.40) | 1.60 (0.27) | 2.21 (0.27) | 1.67 (0.28) | | 1.33 (0.26) | 1.81 (0.32) | 1.88 (0.87) | 2.09 (0.57) |
| *Manufacturer* |  |  |  |  | |  |  |  |  |
| GE | 2.33 (0.29) | 1.74 (0.28) | 2.12 (0.18) | 1.69 (0.29) | | 1.38 (0.14) | 1.77 (0.27) | 1.72 (0.33) | 2.05 (0.41) |
| Philips | 2.44 (0.49) | 1.63 (0.28) | 2.19 (0.28) | 1.66 (0.26) | | 1.38 (0.31) | 1.89 (0.41) | 2.31 (1.25) | 2.22 (0.68) |
| Siemens | 2.56 (0.48) | 1.69 (0.28) | 2.32 (0.28) | 1.75 (0.28) | | 1.35 (0.23) | 1.86 (0.25) | 1.90 (0.81) | 2.02 (0.59) |

Table S3: Mean volume, mean volume similarity, mean Dice coefficient and mean 95% Hausdorff distance for CN subjects from the ADNI HarP dataset, split across field strength and scanner manufacturer

| Group: CN | **FreeSurfer** | | **SynthSeg** | | **FastSurfer** | | **FIRST** | | **Hippodeep** | | | **FreeSurfer-Subfields** | | **HippUnfold** | | **HSF** | |
| --- | --- | --- | --- | --- | --- | --- | --- | --- | --- | --- | --- | --- | --- | --- | --- | --- | --- |
| **Volume (mm^3^)** | | | | | | | | | |  |  |  |  |  |  |  |  |
| *Field Strength* |  | |  | |  | |  | |  | | |  | |  | |  | |
| 1.5T | 3,739 (579) | | 3,960 (515) | | 3,836 (567) | | 3,694 (488) | | 3,020 (515) | | | 3,294 (483) | | 2,608 (424) | | 4,524 (540) | |
| 3T | 3,784 (561) | | 3,872 (553) | | 3,895 (574) | | 3,718 (577) | | 2,942 (517) | | | 3,231 (439) | | 2,572 (363) | | 4,339 (844) | |
| *Manufacturer* |  | |  | |  | |  | |  | | |  | |  | |  | |
| GE | 3,609 (448) | | 3,877 (429) | | 3,714 (411) | | 3,665 (409) | | 2,904 (353) | | | 3,131 (304) | | 2,512 (265) | | 4,117 (844) | |
| Philips | 3,889 (727) | | 3,949 (715) | | 3,998 (748) | | 3,721 (665) | | 3,046 (713) | | | 3,344 (615) | | 2,643 (529) | | 4,770 (670) | |
| Siemens | 3,769 (461) | | 3,929 (415) | | 3,881 (469) | | 3,732 (498) | | 3,000 (410) | | | 3,317 (394) | | 2,616 (348) | | 4,422 (369) | |
| **Volume Similarity** | | | | | | | | | |  |  |  |  |  |  |  |  |
| *Field Strength* |  | |  | |  | |  | |  | | |  | |  | |  | |
| 1.5T | 0.16 (0.08) | | 0.22 (0.08) | | 0.18 (0.07) | | 0.15 (0.11) | | -0.06 (0.08) | | | 0.03 (0.09) | | -0.20 (0.06) | | 0.35 (0.08) | |
| 3T | 0.18 (0.08) | | 0.21 (0.09) | | 0.22 (0.08) | | 0.17 (0.09) | | -0.06 (0.08) | | | 0.04 (0.08) | | -0.19 (0.08) | | 0.31 (0.30) | |
| *Manufacturer* |  | |  | |  | |  | |  | | |  | |  | |  | |
| GE | 0.16 (0.08) | | 0.24 (0.08) | | 0.20 (0.08) | | 0.18 (0.09) | | -0.05 (0.07) | | | 0.03 (0.08) | | -0.18 (0.06) | | 0.27 (0.34) | |
| Philips | 0.16 (0.09) | | 0.18 (0.10) | | 0.19 (0.08) | | 0.12 (0.10) | | -0.09 (0.10) | | | 0.01 (0.10) | | -0.22 (0.08) | | 0.37 (0.15) | |
| Siemens | 0.19 (0.06) | | 0.23 (0.06) | | 0.22 (0.06) | | 0.18 (0.10) | | -0.04 (0.06) | | | 0.06 (0.06) | | -0.18 (0.06) | | 0.35 (0.07) | |
| **Dice Coefficient** | | | | | | | | | |  |  |  |  |  |  |  |  |
| *Field Strength* |  | |  | |  | |  | |  | | |  | |  | |  | |
| 1.5T | 0.73 (0.03) | | 0.82 (0.03) | | 0.74 (0.03) | | 0.82 (0.03) | | 0.83 (0.03) | | | 0.73 (0.02) | | 0.77 (0.03) | | 0.78 (0.05) | |
| 3T | 0.73 (0.03) | | 0.82 (0.03) | | 0.74 (0.02) | | 0.82 (0.03) | | 0.84 (0.02) | | | 0.74 (0.02) | | 0.78 (0.02) | | 0.76 (0.12) | |
| *Manufacturer* |  | |  | |  | |  | |  | | |  | |  | |  | |
| GE | 0.73 (0.03) | | 0.81 (0.03) | | 0.74 (0.03) | | 0.82 (0.03) | | 0.83 (0.03) | | | 0.73 (0.03) | | 0.78 (0.02) | | 0.76 (0.14) | |
| Philips | 0.74 (0.02) | | 0.82 (0.03) | | 0.74 (0.02) | | 0.82 (0.03) | | 0.84 (0.02) | | | 0.74 (0.02) | | 0.77 (0.03) | | 0.78 (0.07) | |
| Siemens | 0.73 (0.02) | | 0.82 (0.02) | | 0.74 (0.03) | | 0.82 (0.02) | | 0.83 (0.02) | | | 0.74 (0.03) | | 0.78 (0.02) | | 0.78 (0.05) | |
| **95% Hausdorff Distance (mm)** | | | | | | | | | |  |  |  |  |  |  |  |  |
| *Field Strength* |  | |  | |  | |  | |  | | |  | |  | |  | |
| 1.5T | 2.13 (0.14) | | 1.56 (0.21) | | 2.08 (0.14) | | 1.64 (0.34) | | 1.31 (0.20) | | | 1.82 (0.25) | | 1.80 (0.35) | | 2.02 (1.01) | |
| 3T | 2.19 (0.25) | | 1.51 (0.21) | | 2.05 (0.17) | | 1.65 (0.30) | | 1.28 (0.21) | | | 1.68 (0.28) | | 1.64 (0.31) | | 3.21 (5.86) | |
| *Manufacturer* |  |  | |  | |  | |  | | |  | |  | |  | |  |
| GE | 2.11 (0.12) | | 1.54 (0.22) | | 2.02 (0.16) | | 1.61 (0.30) | | 1.31 (0.20) | | | 1.76 (0.24) | | 1.71 (0.28) | | 3.68 (6.94) | |
| Philips | 2.17 (0.22) | | 1.54 (0.19) | | 2.11 (0.14) | | 1.67 (0.39) | | 1.30 (0.19) | | | 1.77 (0.28) | | 1.86 (0.42) | | 2.02 (0.83) | |
| Siemens | 2.20 (0.24) | | 1.53 (0.23) | | 2.06 (0.16) | | 1.67 (0.27) | | 1.28 (0.22) | | | 1.73 (0.29) | | 1.61 (0.26) | | 2.07 (1.25) | |
